# Editorial: The organs of sensibility: multimodal sensing within the microbiota-gut-brain axis and how it drives physiology, behavior and perception

**DOI:** 10.3389/fnins.2024.1380039

**Published:** 2024-04-15

**Authors:** Rim Hassouna, Lukas Van Oudenhove, Boris Le Nevé, Ann-Sophie Barwich

**Affiliations:** ^1^Danone Nutricia Research, Danone Global Research & Innovation Center, Gif-sur-Yvette, France; ^2^Translational Research Center for Gastrointestinal Disorders, Katholieke Universiteit Leuven, Leuven, Belgium; ^3^Laboratory for Brain-Gut Axis Studies, Translational Research Center for Gastrointestinal Disorders, Katholieke Universiteit Leuven, Leuven, Belgium; ^4^Leuven Brain Institute, Katholieke Universiteit Leuven, Leuven, Belgium; ^5^Consultation-Liaison Psychiatry, University Psychiatric Centre Katholieke Universiteit Leuven Campus Gasthuisberg, Leuven, Belgium; ^6^Cognitive and Affective Neuroscience Laboratory, Department of Psychological and Brain Sciences, Dartmouth College, Hanover, NH, United States; ^7^Cognitive Science Program, Indiana University, Bloomington, IN, United States; ^8^Department of History and Philosophy of Science and Medicine, Indiana University, Bloomington, IN, United States

**Keywords:** perception, microbiota-gut-brain axis, sensing, behavior, physiology

An exhilarating pace has been set in the exploration of the microbiota-gut-brain axis (hereafter referred to with the acronym MGBA). A growing body of research has laid bare the profound impact of our gut on the brain's workings, neural dynamics, and our behaviors (Valles-Colomer et al., 2019; Radjabzadeh et al., 2022). However the converse is equally true. Our emotional states and perceptions also impact gastrointestinal functions, highlighting a complex, bidirectional relationship that deepens our insight into mind-body connections (Cryan et al., 2019; Brushett et al., 2023).

Still, we are confronted with a critical gap in our understanding of the cellular and molecular mechanisms driving the gut-brain communication axis, especially when it comes to humans. A significant roadblock in our quest for understanding is the scarcity of non-invasive tools tailored for clinical research. This shortage means we are mostly leaning on data from animal models and translational studies, with significant results remaining scarce (Kelly et al., 2017; Margolis et al., 2021).

Navigating this two-way street reveals a maze of interacting components, adding layers of complexity and challenge. It is becoming obvious that cracking the code of the MGBA's varied communication mechanisms demands a deeper grasp of how the gut senses its surroundings. Interoceptive signals play a key role in a host of functions like energy balance, nutrient detection, hunger and satiety cues, gut stability, and the interaction between host and microbiota. Just as crucial is understanding how emotions, stress, and the broader external environment influence the digestive system.

Our goal was to focus on the various ways signals are detected, not just in the brain but also in the gut. This Research Topic includes an original study, a theoretical piece, a review, and a perspective, each offering different insights and fresh outlooks on these issues. These pieces cover three key areas: the immune system, mental states, and the digestive system.

The immune system plays a pivotal role in the dialogue within the MGBA. How the immune system affects visceral pain is the focus of Pawlik et al.'s investigation. Specifically, they explore how pro-inflammatory cytokines circulating in the blood of healthy people affect individual differences in sensing internal visceral signals. Mental states affecting the MGBA is the central focus of the following two pieces. Han et al. give us a comprehensive overview of how the MGBA connects to emotion-related disorders, with an eye on depression. They make the case for deeper exploration into the MGBA's workings to improve our investigation and treatment of gut-brain disorders. Within the same line of research and looking at MGBA through the prism of short-chain fatty acids, Mansuy-Aubert et al. illuminate how these fatty acids could revolutionize human health in ways that affect metabolic and psychological wellness.

Finally, how should we frame our understanding of the gut within the gut-brain axis? Boem et al. expand on the notion that to fully understand cognition and perception, we must view the digestive system as a multifaceted organ/ecosystem, integrating the enteric nervous system, endocrine system, immune system, microbiota, as well as gut tissue and mucosal structures.

Taken together, this accumulation of knowledge showcases the possibility of new investigative pathways–pathways leading to enhanced well-being, sharper diagnostic processes, and targeted treatments for the intricate disorders profoundly linked to the workings of the MGBA. With a blend of hope and anticipation, we hope these perspectives will meet your interest.

## Author contributions

RH: Writing – original draft, Writing – review & editing. LV: Writing – original draft, Writing – review & editing. BL: Writing – original draft, Writing – review & editing. A-SB: Writing – original draft, Writing – review & editing.
